# Heterotopic gastric mucosa in the second part of the duodenum causing hemorrhage in a child: a case report and literature review

**DOI:** 10.3389/fped.2025.1641000

**Published:** 2025-09-25

**Authors:** Qin Huang, Lin Liu, Lu Zhan, Yuanlu Zhu, Quanmin Deng

**Affiliations:** Department of Pediatrics, People’s Hospital of Deyang City, Deyang, Sichuan, China

**Keywords:** heterotopic gastric mucosa, the second part of the duodenum, endoscopy, gastrointestinal bleeding, heterotopic gastric mucosa imaging

## Abstract

Heterotopic gastric mucosa (HGM) refers to the presence of histologically normal gastric tissue in ectopic anatomical locations. HGM is commonly found in the esophagus, duodenal bulb, and Meckel's diverticulum, but its occurrence in the second part of the duodenum is exceedingly rare. HGM may present with gastrointestinal bleeding, obstruction, or intussusception, with painless bleeding often being the initial symptom, which may progress to hemorrhagic shock. We report a case of a child with HGM in the anatomical part of the duodenum (second part) complicated by hemorrhage, managed at Deyang People's Hospital, along with a literature review.

## Introduction

Heterotopic gastric mucosa (HGM) is a rare congenital anomaly characterized by the presence of gastric tissue outside the stomach. While most frequently observed in the gastrointestinal tract, HGM has been reported in diverse locations, including the nasopharynx, biliary tract, pancreas, and anorectal region (1–4). However, involvement of the second part of the duodenum is exceptionally uncommon. HGM is often asymptomatic but may manifest with complications such as bleeding, obstruction, or intussusception. The reported incidence of HGM in the esophagus is relatively high, ranging from 0.1% to 13.8%. The duodenum is also an occasional site of lesions. Herein, we present a pediatric case of HGM in the second part of the duodenum presenting with a massive hemorrhage and review its clinical features, diagnostic approaches, and management.

## Case report

### Clinical history

A 4-year-and-5-month-old boy from Sichuan, China, was admitted to our pediatric ward due to passing black stools twice. The child's mother reported that approximately half a day before admission, the child passed two episodes of black, loose stools without any obvious cause. The stools contained no mucus or fresh red or dark red blood, and were not accompanied by vomiting, abdominal distension, or abdominal pain. The child exhibited a pale complexion and nail beds, appeared lethargic, had no fever, and did not produce tea-colored urine. The laboratory test results upon admission were hemoglobin (Hb) level = 5.5 g/dL, hemocrit (Hct) = 17.20%, and mean corpuscular volume (MCV) = 80.3 fL, and a fecal occult blood test was positive. There was no history of vomiting, abdominal pain, medication use, or allergies and no family history of bloody stools. The preliminary diagnosis upon admission was gastrointestinal bleeding with severe anemia.

The patient presented as an apparently healthy male child with age-appropriate body proportions. On admission, the following vital signs were recorded: body temperature: 36.6 °C, heart rate: 142 beats per min, respiratory rate: 35 breaths per min, and capillary refill time: less than 3 s. The physical examination revealed notable findings, including pale sclera and peripheral acrocyanosis (pallor of the nail beds). Abdominal palpation demonstrated no evidence of tenderness or rebound tenderness. A digital rectal examination identified the presence of black, tarry stools, consistent with melena. No additional pathological findings were observed during the comprehensive physical examination.

### Diagnostic evaluation

**Figure 1:**
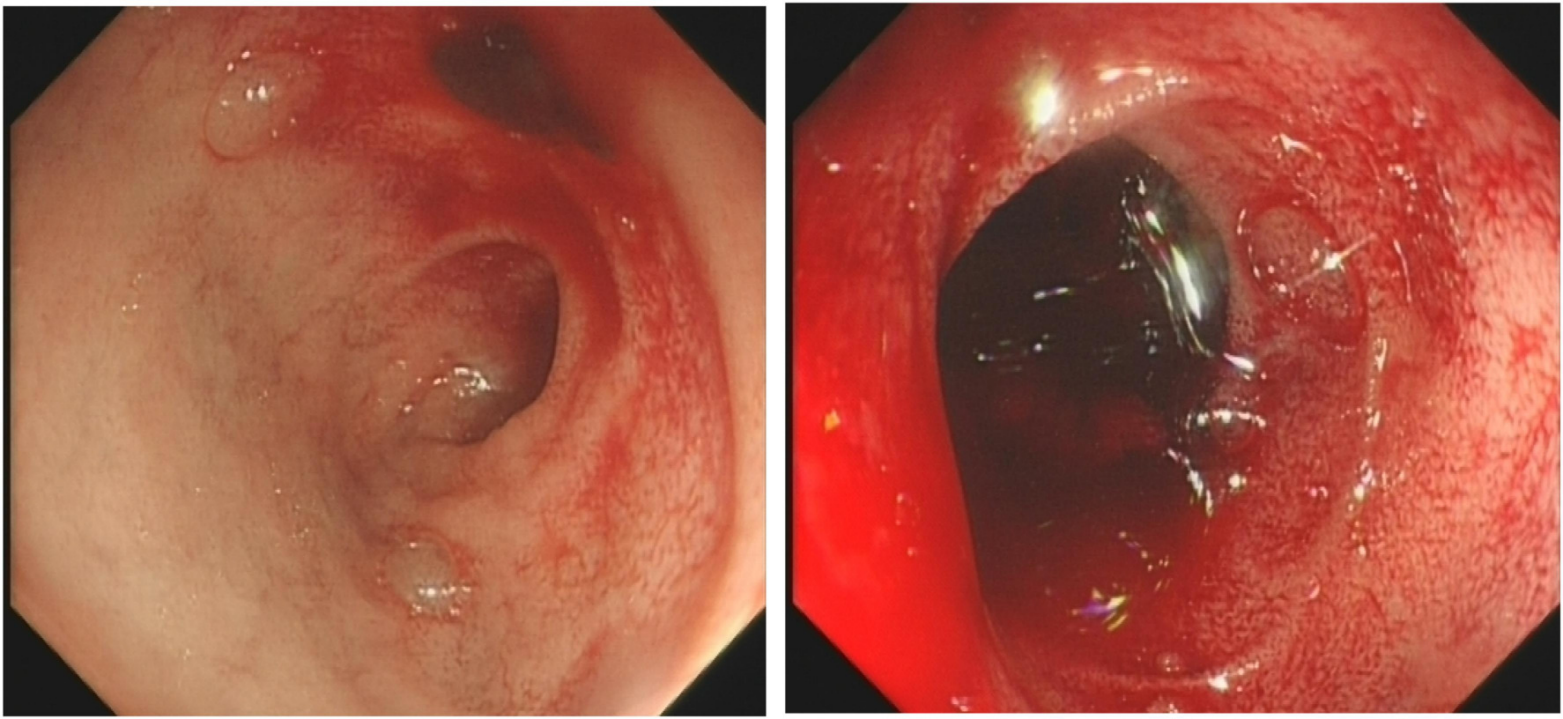
Initial endoscopy showing blood and clots in the duodenal bulb.

Due to severe anemia indicated by the pre-admission complete blood count (CBC), ABO-identical leukocyte-reduced packed red blood cells (two units) were transfused immediately upon admission to correct the anemia. Concurrently, management included nothing by mouth (NPO), gastric acid suppression with omeprazole, hemostatic measures, fluid resuscitation, and supportive care. After the blood transfusion, the child's hemoglobin level rose to 107 g/L, and coagulation function tests showed no abnormalities. After the patient’s hemodynamics stabilized, an upper gastrointestinal endoscopy was performed within 48 h of symptom onset (Figure 1), which revealed fresh blood and clots in the duodenal bulb, with significant fresh blood in the post-bulbar region. The field of view was limited, and no definitive bleeding site was identified. Upper gastrointestinal endoscopy is the preferred method for diagnosing upper gastrointestinal bleeding, with a positivity rate of 80%–90% when performed within 24–48 h of bleeding. Due to significant intraoperative bleeding and poor visibility, endoscopic hemostasis was not performed.

**Figure 2:**
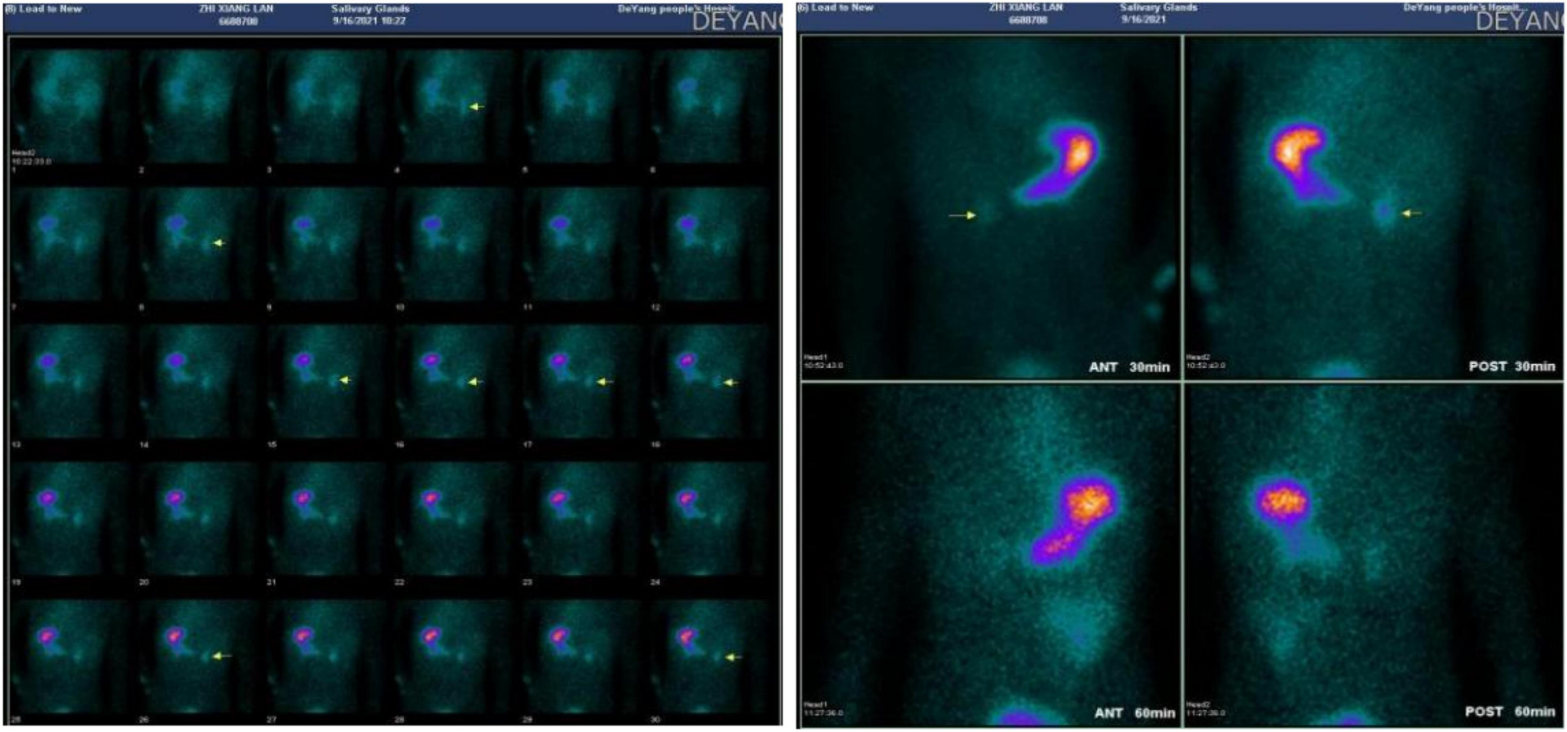
99mTcO4− scintigraphy revealing ectopic gastric mucosa in the second part of the duodenum.

The next day, the child passed two episodes of reddish-brown watery stools, and a repeat hemoglobin test showed a drop to 63 g/L. Therefore, we continued to transfuse two units of leukocyte-reduced suspended red blood cells, added somatostatin to enhance hemostasis, and administered acid-suppressing and fluid replacement therapy. An enhanced abdominal CT scan was performed, which showed minimal fluid accumulation in the jejunum and ileum without significant dilation, no thickening or abnormal enhancement of the intestinal wall, and no obvious abnormalities in the corresponding mesenteric arteries and veins. Since the abdominal CT failed to identify a definitive bleeding site, 99TcmO4− scintigraphy for ectopic gastric mucosa (EGM) was considered to locate the bleeding source. The 99TcmO4− scintigraphy (Figure 2) revealed ectopic gastric mucosa in the second part of the duodenum, ruling out the possibility of lower gastrointestinal bleeding.

**Figure 3:**
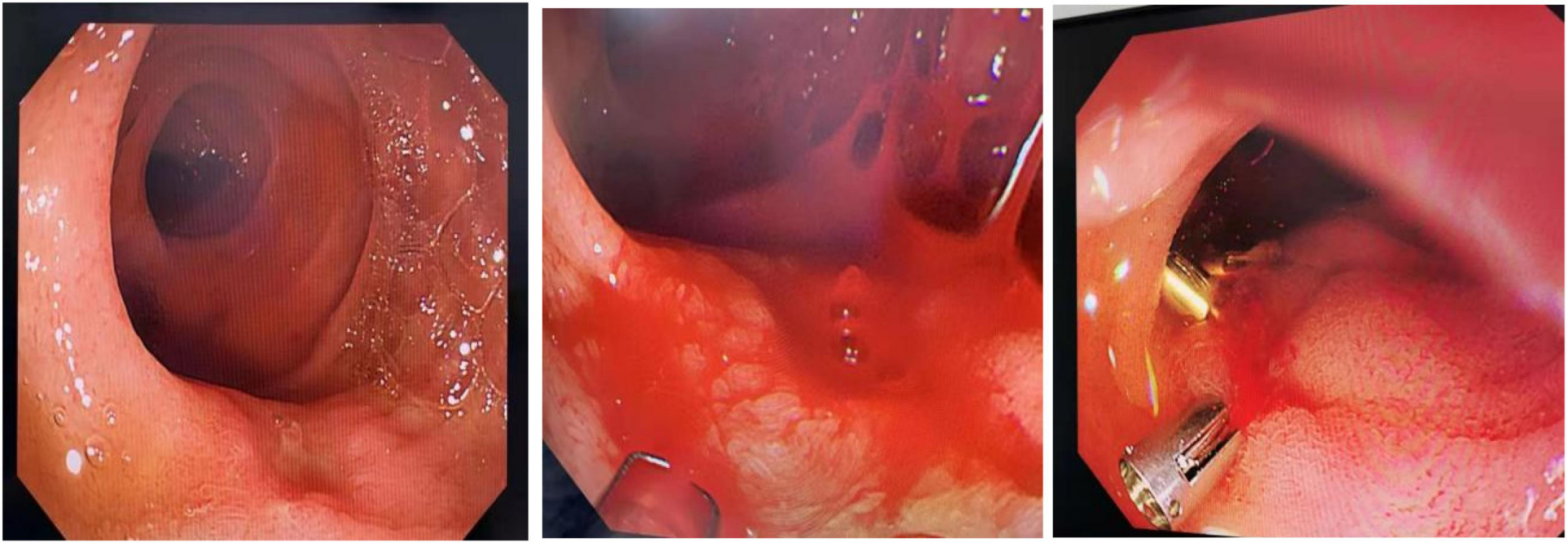
Repeat endoscopy demonstrating the ulcer with active bleeding, which was managed via titanium clip placement.

A repeat upper gastrointestinal endoscopy (Figure 3) showed an ulcer in the second part of the duodenum covered with white exudate, with a visible vessel stump on the surface exhibiting intermittent active bleeding. During the procedure, three titanium clips were used to close the vessel, and no further active bleeding was observed at the ulcer site.

## Treatment and follow-up

After another transfusion of one unit of leukocyte-reduced suspended red blood cells, the patient’s hemoglobin level rose to 134 g/L. The child was observed for 2 days and then started on a liquid diet, subsequently passing yellow stools without mucus or blood streaks. The final diagnosis was revised to ectopic gastric mucosa in the second part of the duodenum with bleeding and severe anemia. The child recovered fully after 1 week of treatment and was discharged. Follow-up to date has shown no recurrence of bleeding, and follow-up gastroscopy 6 months later revealed no significant abnormalities in the mucosa of the second part of the duodenum. The entire treatment process was also highly appreciated by the child's parents.

## Discussion

HGM is rare and is most often found in the duodenal bulb, esophagus, or Meckel's diverticulum (5–8). The molecular pathogenesis has not been fully elucidated. Studies have shown that mutations in *CDX2* (regulating intestinal differentiation) and *SOX2* (maintaining stem cell properties) lead to gastric-type differentiation, while activation of the Wnt/β-catenin pathway promotes gastric mucosal proliferation and gastric acid secretion induces ulceration (9, 10). Ectopic gastric mucosa secretes acid and pepsin, leading to mucosal ulceration and bleeding, as seen in this case. While HGM in the second part of the duodenum is rare, our patient's presentation aligns with reports of painless bleeding as the primary symptom.

### Advancements in diagnostic modalities

The diagnostic techniques for duodenal EGM include endoscopy, nuclear scintigraphy, and SPECT/CT, each with its own advantages and applicable scenarios. Clinicians should select the appropriate diagnostic strategy based on the patient's specific condition. Recent studies have shown that among five SPECT/CT examinations, one case of duodenal diverticulum HGM was diagnosed solely by SPECT/CT, one case with inconclusive results was misdiagnosed as colonic bleeding, and another case had its lesion clearly localized by SPECT/CT. Thus, SPECT/CT appears to outperform conventional planar imaging in diagnosing ectopic gastric mucosa, potentially reducing indeterminate and false-positive results, though further validation is needed. Therefore, the diagnosis of HGM relies on a multimodal approach combining endoscopy, histology, and nuclear medicine. Conventional endoscopy may miss flat or ulcerative lesions, whereas 99mTcO4− nuclear scintigraphy (by detecting functional gastric mucosa) demonstrates an 84% sensitivity for Meckel's diverticulum HGM (8, 11–14). However, bleeding or anemia may lead to false-negative results, necessitating endoscopic evaluation (13). In the active bleeding phase, 99mTcO4− nuclear scintigraphy should be prioritized (detection rate of 45%, specificity of 90.9%). In the non-active bleeding phase, SPECT/CT provides more precise lesion localization than conventional planar imaging (14).

### Special considerations in duodenal diverticular bleeding

HGM is often overlooked because of the following reasons: anatomically concealed location: HGM in the second part of the duodenum is frequently missed during conventional endoscopy due to the complex anatomical structures (e.g., compression by the superior mesenteric artery); non-specific symptoms: painless bleeding and anemia may be misdiagnosed as iron-deficiency anemia or functional dyspepsia; limitations of endoscopy: flat lesions or ulcer margins may require chromoendoscopy (e.g., indigo carmine) for accurate identification. Duodenal diverticular bleeding, a rare cause of upper gastrointestinal hemorrhage, can present as life-threatening hypovolemic shock, as reported in the literature. This case also posed significant challenges in localizing the bleeding source. After multiple endoscopic examinations (one colonoscopy and three esophagogastroduodenoscopies), the bleeding site was finally identified within a diverticulum of the third portion of the duodenum. Endoscopic hemostasis successfully controlled the bleeding, highlighting its critical role in acute management.

Similar to this case, when emergency painless gastrointestinal bleeding occurs and initial endoscopy fails to identify the bleeding site, 99TcmO4− scintigraphy for ectopic gastric mucosa (with a detection rate of 45% and specificity of 90.9%) can be performed during active bleeding. In this case, the scan ultimately revealed ectopic gastric mucosa in the second part of the duodenum, and repeat endoscopic hemostasis led to no further bleeding episodes. Long-term follow-up is essential, as recurrent bleeding or malignant transformation, though rare, has been reported (15–18).

### Differential diagnosis of gastrointestinal bleeding in children

The differential diagnosis of gastrointestinal bleeding in children requires a comprehensive consideration of various etiologies, including ectopic gastric mucosa, duodenal ulcers, inflammatory bowel disease, and intestinal polyps. These conditions exhibit significant differences in clinical manifestations, diagnostic strategies, and treatment approaches. Accurately distinguishing ectopic gastric mucosa from other causes is crucial for early diagnosis and effective treatment. For children with unexplained gastrointestinal bleeding, the possibility of ectopic gastric mucosa should be considered, and targeted examinations should be conducted.

## Conclusion

This case highlights HGM as a rare but critical etiology of pediatric gastrointestinal bleeding. Integrating molecular diagnostics (e.g., CDX2/SOX2 profiling) with advanced diagnostic modalities optimizes the patient’s outcomes. Future studies should validate non-invasive biomarkers, such as pepsinogen ratios, in risk stratification. This case underscores the importance of considering HGM in children presenting with unexplained gastrointestinal bleeding. Multi-channel diagnosis, Including endoscopy, SPECT/CT and scintillation imaging. In cases of active bleeding where a single endoscopic examination fails to identify the bleeding site, non-invasive imaging with 99mTcO4− scintigraphy for heterotopic gastric mucosa can be performed, thereby avoiding the need for exploratory laparotomy. Endoscopic therapy offers a safe and effective treatment option, though long-term follow-up is warranted.

## Data Availability

The original contributions presented in the study are included in the article/Supplementary Material, further inquiries can be directed to the corresponding authors.

## References

[1] Wang JQ Xiang LZ Pan XL Zhang CS Yu XY Hou XH. Clinical, endoscopic, and histopathological characteristics of heterotopic gastric mucosa in the upper esophagus. Chin J Dig Endosc. (2022) 39(9):695–700. 10.3760/cma.j.cn321463-20210413-00245

[2] Meng Y Zhou YH Li P. Clinical characteristics and endoscopic diagnosis of heterotopic gastric mucosa in the duodenum. J Cap Med Univ. (2018) 39(5):721–5. 10.3969/j.issn.1006-7795.2018.05.018

[3] Iacopini F Gotoda T Elisei W Rigato P Montagnese F Saito Y Heterotopic gastric mucosa in the anus and rectum: first case report of endoscopic submucosal dissection and systematic review. Gastroenterol Rep (Oxf). (2016) 4(3):196–205. 10.1093/gastro/gow006 27103738 PMC4976682

[4] Ott L O’Neill J Cameron D Callahan MJ Grover A Fox VL Triple gallbladder with heterotopic gastric mucosa: a case report. BMC Pediatr. (2022) 22(1):52. 10.1186/s12887-022-03122-7 35057772 PMC8772126

[5] Kiba T Kotoh N Tsuboi M. Rare presentation of annular and polypoid heterotopic gastric mucosa in duodenum. JGH Open. (2020) 5(2):312–3. 10.1002/jgh3.12434 33553673 PMC7857297

[6] Tuzun A Polat Z Kilciler G Turan I Kilic A Ozcan A Evaluation for *Helicobacter pylori* in Meckel’s diverticulum by using real-time PCR. Dig Dis Sci. (2010) 55(7):1969–74. 10.1007/s10620-009-0958-2 19714464

[7] Murshed KA Khawar M Petkar M. Heterotopic gastric mucosa in the ileum: a rare cause for intussusception in adults. Case Rep Gastroenterol. (2020) 14(3):609–14. 10.1159/000509504 33362448 PMC7747077

[8] Chen XL Wu YL Li XQ Liu W Wang Y. Diagnosis and complications of Meckel’s diverticulum in children: a clinical analysis. J Clin Pediatr Surg. (2017) 16(1):60–4. 10.3969/j.issn.1671-6353.2017.01.014

[9] Voutsadakis IA. Gastric adenocarcinomas with CDX2 induction show higher frequency of TP53 and KMT2B mutations and MYC amplifications but similar survival compared with cancers with no CDX2 induction. J Clin Med. (2024) 13(24):7635. 10.3390/jcm13247635 39768557 PMC11727917

[10] Singh H Seruggia D Madha S Saxena M Nagaraja AK Wu Z Transcription factor-mediated intestinal metaplasia and the role of a shadow enhancer. Genes Dev. (2022) 36(1–2):38–52. 10.1101/gad.348983.121 34969824 PMC8763054

[11] Jin S Wang RH Liu Y Han XM Xue MM Ruan Q Clinical value of SPECT/CT imaging in diagnosing heterotopic gastric mucosa. Chin J Nucl Med Mol Imaging. (2016) 36(3):229–31. 10.3760/cma.j.issn.2095-2848.2016.03.006

[12] Long Y Jin HB Yu Y Tang M Li XL Li B. Diagnostic value of 99mTcO4- ectopic gastric mucosa imaging for Meckel’s diverticulum. J Chin Pract Diagn Therapy. (2019) 33(5):491–3. 10.13507/j.issn.1674-3474.2019.05.022

[13] Huang XS Wu FF Wang WD Shi J Xie F Bai L Analysis of preoperative diagnostic methods for 157 symptomatic Meckel’s diverticulum cases. Mod Dig Interv. (2021) 26(5):565–70. 10.3969/j.issn.1672-2159.2021.05.005

[14] Koç ZP Özcan PP Tuncel F İsbir C Usta Y. SPECT/CT in the diagnosis of ectopic gastric mucosa—Meckel’s diverticulum. World J Nucl Med. (2024) 23(3):176–9. 10.1055/s-0044-1787719 39170839 PMC11335389

[15] Sharma M Somani P Janarthanan K Jindal S Prasad R Hari RS. Endoscopic ultrasound of duodenal heterotopic gastric mucosa. Endoscopy. (2017) 49(7):E168–70. 10.1055/s-0043-105569 28525932

[16] Abe T Kato M Kohno S Hamatani S Kawahara Y Isshi K Foveolar gastric metaplasia presenting as a duodenal tumor with an atypical appearance: a case report. J Med Case Rep. (2016) 10(1):355. 10.1186/s13256-016-1163-5 27998304 PMC5175308

[17] Li Y Xing YX Wang Q. Diagnostic value of narrow-band imaging endoscopy for duodenal gastric metaplasia and heterotopic gastric mucosa. Int J Dig Dis. (2018) 38(1):37–41. 10.3969/j.issn.1673534X.2018.01.009

[18] Toyoda F Matsumori T Matsumoto A Kashima H Matsuyama S Ueo T Difficulty in identifying the source of hemorrhage in duodenal diverticula bleeding: a case report. Intern Med. (2025). 10.2169/internalmedicine.5222-24 40159163 PMC12549038

